# Earthworm (*Eisenia andrei*) Avoidance of Soils Treated with Cypermethrin

**DOI:** 10.3390/s111211056

**Published:** 2011-11-28

**Authors:** Ana Paula A. de Sousa, Mara M. de Andréa

**Affiliations:** 1 Prefeitura Municipal de São Paulo, Av. Prof. João Batista Conti 331, CEP 08255-210, São Paulo, SP, Brazil; E-Mail: ana_pas1@yahoo.com.br; 2 Instituto Biológico, Lab. de Ecologia de Agroquímicos, Av. Conselheiro Rodrigues Alves 1252, CEP 04014-002, São Paulo, SP, Brazil

**Keywords:** pesticide, pyrethroid, soil parameters, pesticide formulation

## Abstract

The pyrethroid insecticide cypermethrin is used for agricultural and public health campaigns. Its residues may contaminate soils and the beneficial soil organisms, like the earthworms, that may ingest the contaminated soil particles. Due to its ecological relevance, earthworms *Eisenia andrei/fetida* have been used in different ecotoxicological tests. The avoidance of soils treated with cypermethrin by compost worms *Eisenia andrei* was studied here as a bioindicator of the influence of treatment dosage and the pesticide formulation in three different agricultural soils indicated by the Brazilian environmental authorities for ecotoxicological tests. This earthworms’ behavior was studied here as a first attempt to propose the test for regulation purposes. The two-compartment test systems, where the earthworms were placed for a two-day exposure period, contained samples of untreated soil alone or together with soil treated with technical grade or wettable powder formulation of cypermethrin. After 48 h, there was no mortality, but the avoidance was clear because all earthworms were found in the untreated section of each type of soil (p < 0.05). No differences were found by the Fisher’s exact test (p ≤ 1.000) for each soil and treatment, demonstrating that the different soil characteristics, the cypermethrin concentrations and formulation, as well as the smaller amounts of soil and earthworms did not influence the avoidance behavior of the earthworms to cypermethrin. The number and range of treatments used in this study do not allow a detailed recommendation of the conditions applied here, but to the best of our knowledge, this is the first reported attempt to identify the avoidance of pesticide treated tropical soils by earthworms.

## 1. Introduction

Agricultural and domestic uses of pesticides introduce agrochemicals to the soil environment because the synthetic and xenobiotic compounds are applied directly to the soil or some residues from the foliar or aerial applications reach the soil environment where they may persist [1,2]. Soil pollution has important consequences to all forms of life and to the food, water and air quality because the soil is the source of water and nutrients to plants and animals, as well as the habitat for many species that are important to the nutrient cycling and availability [3,4].

The soil environment contains many organisms from different trophic levels that may be all exposed even if only a few members of the soil food web are exposed. If the contaminated organisms belong to the lower trophic levels, the probability of a widespread contamination and the possibility of bioaccumulation and biomagnification of the contaminants along the food chain are even higher because they are food for several other organisms in the web, acting as a route to contaminants transference [5]. Earthworms are one of these groups of animals that belong to the very complex soil food web. They may be exposed to pesticide residues that are directly applied or reach the soil, either because this is their main source of food or because the contaminants may be absorbed by their body surface [6,7]. Due to their ecological relevance, earthworms have been used as bioindicators and, as they are also biosensors of sublethal concentrations, they could serve as a warning sign for the early effects of soil contamination [6].

Despite the fact that it has been noted that *Eisenia* is a less sensitive earthworm species [8–10], *Eisenia fetida* and *E. andrei* are the commonest bioindicators of soil contamination with pesticides, mainly because they are easy to rear and maintain in laboratory conditions [6,7]. The determination of the bioaccumulation factor of the contaminants (BAF) and the bioassays that assess the effects of contaminants on earthworms’ reproductive parameters have been proved sensitive [11], but the behavioral changes have been pointed as useful to detect adverse effects generated by their exposition to sublethal doses [12]. These behavioral changes due to the presence of contaminants in soils can be detected, for example, by the avoidance test [13,14]. The main advantages of using the avoidance behavior to evaluate the ecological risks are the short duration of the test—just 48 h—and its easiness of set up [15].

However, so far, the government authorities have not included the avoidance behavior of the compost worms *Eisenia fetida/andrei* as one of the required tests for regulation of pesticide molecules; but, the use of natural soils as substrates for bioaccumulation of chemicals in terrestrial organisms was recently adopted as a guideline [16]. Artificial soils currently used [17,18] may give universal data, but they may be different in natural agricultural soils [8,19]. Moreover, most tests on the influence of chemicals on the avoidance behavior of earthworms use large amounts of treated soils [17,20], which may present a laboratory pollution problem at the time of disposal.

Using basically the test conditions of the ISO 17512-1 [18] and ABNT NBR ISO 17512-1 [20] guidelines, this work aimed to verify the avoidance of earthworms *Eisenia andrei* to agricultural natural soils indicated by the Brazilian environmental authorities for ecotoxicological tests [21] that were treated with a commercial formulation of cypermethrin, or with the technical grade compound. Smaller amounts of organisms and soils were also used in order to test other meaningful and environmentally friendly conditions for the studies.

## 2. Experimental Setup

It is known that the soil characteristics have great influence on the environmental fate and bioavailability of pesticides [1] and, therefore, some assays with biosensors should be done with natural soils to achieve better understanding of the possibilities of food web contamination in real environments. After application, some pesticide residues may persist in soil leading to high risk exposure of soil organisms to the product. Among them, the earthworms may be in direct contact and ingest the contaminated soil particles. However, by having sensory tubercles on their body surfaces, depending on the pollutant concentration, they can detect and avoid the contaminated soil [10]. The avoidance behavior has been verified for some pesticides in natural soils [22–24], including cypermethrin, but only in artificial soil [25].

The Brazilian Institute of Environment and Natural Resources (IBAMA) has already established three agricultural soils for some ecotoxicity tests (Table 1), whose physical and chemical parameters cover the main causes of differences in pesticide behavior [21]. These soils were here utilized for bioindication of cypermethrin effects on the compost worms *Eisenia andrei*.

As one of the most recent pesticides, the pyrethroid insecticide cypermethrin is widely used in agricultural and public health campaigns due to its efficiency in controlling insects [26]. The recommended doses are from 10 to 75 g (a.i.) ha^−1^ soil [27].

Although cypermethrin is reported to have moderate toxicity to animals and a moderate persistence in soils [28], its residues were found in creeks after agricultural and urban soil applications [29]. It was also found in soils and agricultural products (vegetable and fish samples) [30], meaning that it is persistent and mobile enough to be detected in the soil, water and organisms sometime after the application.

The earthworms’ avoidance behavior towards the three cypermethrin-treated soils was here studied basically according to the ISO 17512-1 [18] and ABNT NBR ISO 17512-1 [20] guidelines, using three replicates of plastic square chambers with 500 mL capacity (13 cm × 13 cm × 5 cm high) for each dose of treatment. The chambers were divided in two equal parts and 100 g of each soil moistened and maintained at 60% WHCmax during the previous week were placed in each side of the chambers. The treatment was applied to the soil in just one side of the chambers (C1, C2 or C3) and the other side remained cypermethrin-untreated—control soil (C0), along with double control chambers (C0–C0). The size of the chambers and the proportion of soil: earthworms were almost the same used by [2] for acute toxicity test in a four day study.

The treatments were 15, 30 and 60 μg technical grade cypermethrin g^−1^ soil, and the double-control were treated with the maximum volume of the acetone solution (360 μL) used in the treatment. The wettable powder cypermethrin was diluted in ethanol and the treatment corresponded to 15, 25 and 35 μg (a.i.) g^−1^ soil, respectively for C1, C2 and C3; the double-control were treated with 5.0 mL of ethanol. The systems remained overnight in fume hood for solvent evaporation, the divider was then removed and six adult earthworms (>300 mg, with clitelum) were placed all together in the slit in the middle of the chambers. The utilized doses were much higher than the recommended ones because, in a separate study (results not reported), the earthworms did not die in contact with up to 340 μg of active ingredient of cypermethrin.

All the chambers were closed with perforated plastic film to allow air circulation, and maintained at approximately 22 °C under continuous light for 48 h. At the end of the test period, the counting of the compost worms was done on each side of the chambers. According to ISO 17512-1 [17], the avoidance to the different soil treatments was calculated by counting the mean number of earthworms in each concentration and compared with the mean number of worms in the untreated control soil. Before the placement of the earthworms in the chambers and after their removal, (3× approximately) 3 g samples of the treated soils were removed for monitoring the pH in order to verify other causes of the earthworms’ behavior.

The amount of earthworms was converted to percentage of avoidance by the following equation: R(%) = [(C − T)/N] × 100, where R = avoidance; C = number of worms in the control (C0) condition; T = number of worms in each dose in the same soil; N = total number of worms [18].

## 3. Experimental Results and Discussion

Although the treatments here used exceed the LC_50_ of cypermethrin (0.054 μg kg^−1^ soil) found by others [2], no matter the dose, no mortality was found, and the distribution of the earthworms in the C0–C0 condition was 50% in each side of the chambers with the soils PV and GM, and 60% and 40% in the chamber with the soil LV, indicating an homogenous distribution. The distribution of the worms found in the double control (C0–C0) was within the range 40%–60%, in the three studied soils, as the recommended validity criteria of the ISO 17512-1 [18].

Conversely, the soils treated with cypermethrin were avoided by the earthworms already at the smaller dose of the technical grade cypermethrin (C1 = 15 μg g^−1^) in the three studied soils (Table 2). Moreover, the Fisher Exact Test indicated that the number of earthworms in the control condition (C0) was significantly larger than in the treated conditions (C1, C2 and C3), in all three soils (p < 0.05). These results clear indicate the earthworms’ avoidance to the soils treated with cypermethrin.

In order to verify if the increase in the concentration of treatments would influence the avoidance behavior, the frequency of earthworms in each concentration was compared two-to-two by the Fisher’s test (C1 × C2; C1 × C3; and C2 × C3), for the three soils. Results demonstrate that there was no significant difference between the percentage of avoidance and the increase of technical grade cypermethrin concentrations in the soils (p from 0.3333 to 1.0000).

The earthworms’ avoidance was also observed when the soils were treated with the wettable powder commercial formulation of cypermethrin (Table 3) because the number of compost worms was always greater in the control condition (p < 0.05) than in all treated soils (C1, C2 and C3). But no mortality was observed in any soil. In the same way, the increase in concentration of the formulated insecticide did not induce an avoidance increase, and the frequency of the worms was not significantly different independently of the concentration increase of the formulated insecticide in the three soils (p from 0.0801 to 1.0000).

As the soil pH remained almost the same as before (Table 1) and after the treatments (LV: 4.6; PV: 6.2, and GM: 4.2), it is possible to conclude that the observed effects were caused by the insecticide cypermethrin, independently of its presentation. Thus, as reported by others with artificial soil [29], the increase in cypermethrin concentration for soil treatments did not influence the earthworms’ avoidance behavior.

## 4. Conclusions

The number and the range of treatments utilized in this study do not allow a detailed recommendation of the conditions here utilized, but to the best of our knowledge, these are the first reported attempt to identify earthworms’ avoidance of tropical soils treated with pesticides. The earthworms *Eisenia andrei* avoided the soils treated with cypermethrin, but there was no dose-related response within the range of concentration tested, independently of being formulated as wettable powder or technical grade. As no mortality was detected, the test conditions may be further studied in order to be indicated as meaningful and environmentally friendly.

## Figures and Tables

**Table 1.** Main properties of the soils used for determining the avoidance of earthworms *Eisenia andrei* to treatments with cypermethrin.

**Soil**	**pH (H_2_O)**	WHCmax * (mL g^−1^ dry soil)	OMC **	**N**	**Clay**	**Silt**	**Sand**
			**–––––––g kg^−1^–––––**		**––––––––%––––––––**		
Typic Hapludox—LV	4.7	0.62	39.6	2.80	63	14	23
Mollic Hapludalf—PV	6.0	0.41	41.3	3.00	48	18	34
Typic Humaquept—GM	4.1	1.43	127.3	6.99	60	33	7

* maximum water-holding capacity;

** organic matter content.

**Table 2.** Avoidance of the earthworms *Eisenia andrei* to three different agricultural soils treated with different concentrations of technical grade cypermethrin.

Soil * Chambers	**Control (C0^#^) × Treatments (C1, C2 and C3)**		
	**C0–C1**	**C0–C2**	**C0–C3**
PV-1	4 2	6 0	6 0
PV-2	5 1	5 1	6 0
PV-3	6 0	6 0	5 1
Total (n) ^§^	15 3	17 1	17 1
R (%) ^†^	66.6	88.8	88.8
LV-1	6 0	6 0	6 0
LV-2	4 2	5 1	5 1
LV-3	6 0	6 0	6 0
Total (n)	16 2	17 1	17 1
R (%)	77.7	88.8	88.8
GM-1	5 1	6 0	6 0
GM-2	6 0	6 0	6 0
GM-3	5 1	6 0	6 0
Total (n)	16 2	18 0	18 0
R (%)	77.7	100	100

* Soils: PV = Mollic Hapludalf, LV = Typic Hapludox, and GM = Typic Humaquept;

# C0 = untreated-control soils, C1, C2, C3 = respectively 15, 30 and 60 μg g^−1^;

§ Total number of worms in each side of the chambers;

† Percentage of avoidance to the contaminated soil: R% = [(C − T)/N] × 100, where C = number of worms in the Control condition, T = number of worms in the treated conditions, N = total number of worms.

**Table 3.** Avoidance of the earthworms *Eisenia andrei* to three different agricultural soils treated with different concentrations of the wettable formulation of cypermethrin.

Soil * Chambers	**Control (C0^#^) × Treatments (C1, C2 and C3)**		
	**C0–C1**	**C0–C2**	**C0–C3**
PV-1	6 0	3 3	6 0
PV-2	4 2	6 0	5 1
PV-3	3 3	5 1	6 0
Total (n) ^§^	13 5	14 4	17 1
R (%) ^†^	44.4	55.5	88.8
LV-1	6 0	6 0	6 0
LV-2	4 2	6 0	6 0
LV-3	6 0	6 0	6 0
Total (n)	16 2	18 0	18 0
R (%)	77.7	100	100
GM-1	4 2	6 0	6 0
GM-2	6 0	5 1	6 0
GM-3	5 1	5 1	6 0
Total (n)	15 3	16 2	18 0
R (%)	66.6	88.8	100

* Soils: PV = Mollic Hapludalf, LV = Typic Hapludox, and GM = Typic Humaquept;

# C0 = untreated-control soils, C1, C2, C3 = respectively 15, 25 and 35 μg g^−1^;

§ Total number of worms in each side of the chambers;

† Percentage of avoidance to the contaminated soil: R%= [(C − T)/N] × 100, where C = number of worms in the Control condition, T = number of worms in the treated conditions, N = total number of worms.
